# Examining the molecular characteristics of glycoside hydrolase family 20 β-N-acetylglucosaminidases with high activity

**DOI:** 10.1080/21655979.2019.1602427

**Published:** 2019-04-13

**Authors:** Rui Zhang, Shujing Xu, Xinyue Li, Xiaowei Han, Zhifeng Song, Junpei Zhou, Zunxi Huang

**Affiliations:** a Engineering Research Center of Sustainable Development and Utilization of Biomass Energy, Ministry of Education, Yunnan Normal University, Kunming, P. R. China; b College of Life Sciences, Yunnan Normal University, Kunming, P. R. China; c Key Laboratory of Yunnan for Biomass Energy and Biotechnology of Environment, Yunnan, Kunming, P. R. China; d Key Laboratory of Enzyme Engineering, Yunnan Normal University, Kunming, P. R. China

**Keywords:** Β-N-acetylglucosaminidase, glycoside hydrolase family 20, catalytic activity, random coil, acidic amino acid

## Abstract

β-N-Acetylglucosaminidases (GlcNAcases) possess many important biological functions and are used for promising applications that are often hampered by low-activity enzymes. We previously demonstrated that most GlcNAcases of the glycoside hydrolase (GH) family 20 showed higher activities than those of other GH families, and we presented two novel GH 20 GlcNAcases that showed higher activities than most GlcNAcases. A highly flexible structure, which was attributed to the presence of to a high proportion of random coils and flexible amino acid residues, was presumed to be a factor in the high activity of GH 20 GlcNAcases. In this study, we further hypothesized that two special positions might play a key role in catalytic activity. The increase in GH 20 GlcNAcase activity might correspond to the increased structural flexibility and substrate affinity of the two positions due to an increase in random coils and amino acid residues, notably acidic Asp and Glu.

## Introduction

β-N-acetylglucosaminidases (GlcNAcases, EC 3.2.1.52) play a role in the hydrolysis of terminal N-acetyl-D-glucosamine residues in N-acetyl-β-D-glucosaminides. These enzymes possess many important biological functions and are involved in a wide range of industrial applications, such as dynamic balancing of cellular O-linked N-acetylglucosamine (GlcNAc) levels[1]; bacterial cell wall recycling[2]; proper assembly of bacterial flagella[3]; bioconversion of chitin waste to sialic acid[4], bioethanol[5], and single-cell protein[6]; and synthesis of biologically important oligosaccharides [7,8].

Chitin is a naturally abundant biomass and nitrogenous organic compound; 10^12^–10^14^ tons of chitin is produced annually, ranking second in worldwide production after lignocellulose[9]. However, chitinous materials exhibit low natural degradation rates that can potentially become hazardous to environments [10,11]. Therefore, there is a growing demand for high-activity chitinases and GlcNAcases for the degradation of chitin in commercial, biotechnological, and environmental uses.

GlcNAcases are widely distributed among various natural sources including animals, plants, insects, bacteria, and fungi, and they belong to glycoside hydrolase (GH) families 3, 20, 73, 84, and 85[12]. However, we previously found that most characterized GlcNAcases showed activities below 1000 μmol min^−1^ mg^−1^ toward *p*-nitrophenyl β-N-acetylglucosaminide (*p*NPGlcNAc) or 4-methylumbelliferyl β-N-acetylglucosaminide (MUGlcNAc), and approximately 70% of the characterized GlcNAcases showed activities below 100 μmol min^−1^ mg^−1^[12].

Protein engineering and mining the genomic data of new organisms for novel enzymes are two methods for potentially obtaining highly active GlcNAcases. We previously revealed that most GlcNAcases of GH 20 showed higher activities than those of other GH families[12], two of which were presented as exhibiting higher activity than most GlcNAcases reported in available literature: JB10Nag from *Shinella* sp. with a specific activity of 538.8 μmol min^−1^ mg^−1^ and a *V*_max_ of 1030 μmol min^−1^ mg^−1^[13], and HJ5Nag from *Microbacterium* sp. with a specific activity of 1773.1 μmol min^−1^ mg^−1^ and a *V*_max_ of 3097 μmol min^−1^ mg^−1^[14]. Furthermore, we analyzed the overall molecular characteristics of GH 20 GlcNAcases and indicated that a highly flexible structure, which was ascribed to a high proportion of random coils as well as flexible amino acid residues, was presumed to be a factor in the high activity of GH 20 GlcNAcases [13,14]. Improvements in the catalytic activity of GH 20 GlcNAcases can be rationally designed based on the general molecular characteristics.

According to the general molecular characteristics guide regarding the catalytic activity of GH 20 GlcNAcases, we further analyzed and compared the molecular characteristics of GH 20 GlcNAcases with various activities in the present study. The aim of this study was to reveal that which random coils and flexible amino acid residues may have a key role in the high activity of GH 20 GlcNAcases.

## Materials and methods

### GlcNAcases and strains

Yunnan Province, China is a source of diverse and novel genetic samples [15,16]. We previously isolated *Shinella* sp. JB10 from the slag collected from a phosphate rock-stacking site and *Microbacterium* sp. HJ5 from the saline soil of an old salt mine located in Yunnan Province [13,14]. Strains JB10 and HJ5 were deposited in the Strains Collection of the Yunnan Institute of Microbiology under registration nos. YMF 3.00678 and YMF 4.00007, respectively. Amino acid sequences of GH 20 GlcNAcases JB10Nag and HJ5Nag can be retrieved from GenBank with accession numbers AQM74372 and ARJ33352, respectively.

Besides JB10Nag and HJ5Nag, five GH 20 GlcNAcases exhibiting different specific activities and *V*_max_ values were selected for bioinformatics analysis in this study. Crystal structures of the five GH 20 GlcNAcases have been resolved and published at the Protein Data Bank (PDB; https://www.rcsb.org/). The GlcNAcase Chb (PDB ID: 1QBA) from *Serratia marcescens* shows the highest activity among reported GlcNAcases to date, with a specific activity of 62 750 μmol min^−1^ mg^−1^ toward *p*NPGlcNAc [17,18]. The *V*_max_ values of the other GlcNAcases are as follows: Hex1 (PDB ID: 3GH4) from *Paenibacillus* sp., 212 μmol min^−1^ mg^−1^ toward *p*NPGlcNAc[19]; HexA (PDB ID: 2GJX) from *Homo sapiens*, 171.7 μmol min^−1^ mg^−1^ toward MUGlcNAc [20,21]; SpHex (PDB ID: 1M01) from *Streptomyces plicatus*, 53.2 μmol min^−1^ mg^−1^ toward MUGlcNAc[22]; and OfHex1 (PDB ID: 3NSM) from *Ostrinia furnacalis* shows a specific activity of 53.2 μmol min^−1^ mg^−1^ toward *p*NPGlcNAc [23,24].

### Bioinformatics analysis

Amino acid sequences of GH 20 GlcNAcases were aligned using Vector NTI 10.3 software (InforMax, Gaithersburg, MD, USA). The structure of HJ5Nag was previously modelled using the homology modelling approach with the SwissModel platform (http://swissmodel.expasy.org/) using SpHex as template, a GMQE score of 0.72, and a sequence identity of 48.9%[14]. The structure of JB10Nag was previously modelled using the threading approach with the I-TASSER platform (http://zhanglab.ccmb.med.umich.edu/I-TASSER/) with a determined C-score of 0.39 and a TM-score of 0.77[13]. Tertiary structures of GH 20 GlcNAcases were visualized using Discovery Studio v2.5 software (Accelrys, San Diego, CA, USA).

## Results

**Figure 1.**
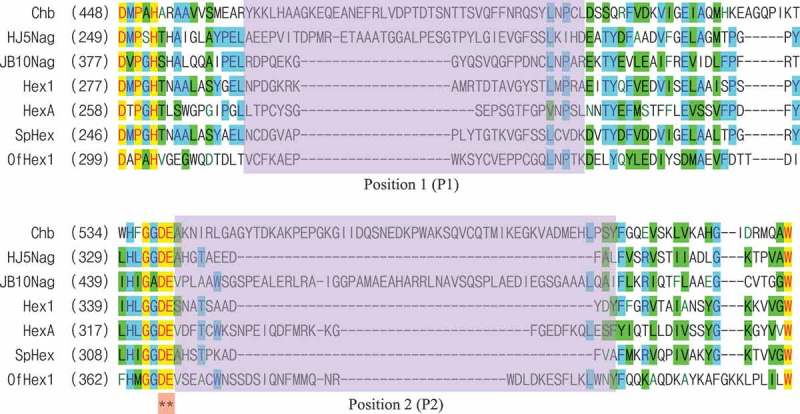
Partial amino acid sequence alignment of GH 20 GlcNAcases. Alignments are given for the following GlcNAcases: Chb (PDB ID: 1QBA; accession no.: AAB03808) from *S. marcescens* [17,18], HJ5Nag (accession no.: ARJ33352) from *Microbacterium* sp [14],. JB10Nag (accession no.: AQM74372) from *Shinella* sp [13],. Hex1 (PDB ID: 3GH4; accession no.: BAI63641) from *Paenibacillus* sp [19],. HexA (PDB ID: 2GJX; accession no.: AAD13932) from *H. sapiens* [20,21], SpHex (PDB ID: 1M01; accession no.: AAC38798) from *S. plicatus*[22], and OfHex1 (PDB ID: 3NSM; accession no.: ABI81756) from *O. furnacalis* [23,24]. Asterisks indicate catalytic residues. The two positions that show great diversity among these GH 20 GlcNAcases are highlighted by the light purple-colored box.

As shown in Figure 1, there were two positions that showed great diversity among the examined GH 20 GlcNAcases. These two positions were located near the catalytic aspartic acid (Asp) residue that functions as a nucleophile/base and the glutamic acid (Glu) residue that functions as a proton donor/acceptor.

Position 1 (P1) of Chb contained 43 amino acid residues, of which 5 were Asp and Glu. P1 of HJ5Nag contained 42 amino acid residues, of which 7 were Asp and Glu. The other GlcNAcases contained fewer than 24 amino acid residues and 3 Asp and Glu (Table 1). Position 2 (P2) of Chb contained 56 amino acid residues, of which 8 were Asp and Glu. P2 of JB10Nag contained 55 amino acid residues, of which 6 were Asp and Glu. The other GlcNAcases contained fewer than 34 amino acid residues and 6 Asp and Glu (Table 1). Thus, Chb, HJ5Nag, and JB10Nag contained many more amino acid residues, including Asp and Glu, than other GlcNAcases in P1 and/or P2.

**Table 1.** Molecular characteristics of GH 20 GlcNAcases.

		P1			P2		
GlcNAcase (PDB ID or accession no.)	Total coil/α-helix	Total amino acids	Amino acids building coils	Asp and Glu	Total amino acids	Amino acids building coils	Asp and Glu
Chb (1QBA)^[17,18]^	1.48	43	19	5	56	17	8
HJ5Nag (ARJ33352)^[14]^	1.49	42	36	7	11	3	3
JB10Nag (AQM74372)^[13]^	1.96	24	20	3	55	27	6
Hex1 (3GH4)^[19]^	1.24	24	20	2	11	4	2
HexA (2GJX)^[20,21]^	1.04	21	6	1	31	9	6
SpHex (1M01)^[22]^	1.22	24	20	2	11	3	1
OfHex1 (3NSM)^[23,24]^	1.12	24	13	2	34	9	5

**Figure 2.**
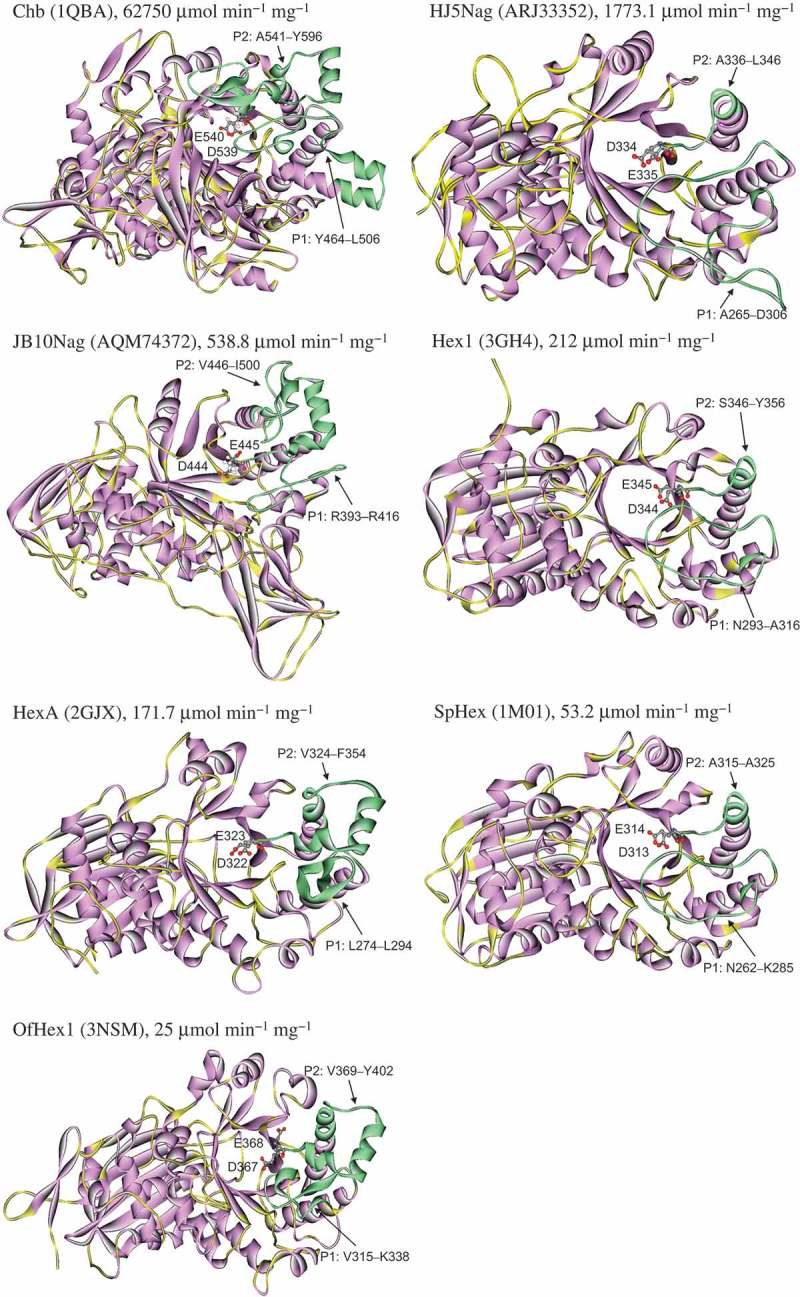
Structures of GH 20 GlcNAcases. Structures are shown for the following GlcNAcases: Chb (PDB ID: 1QBA) from *S. marcescens* [17,18], HJ5Nag (accession no.: ARJ33352) from *Microbacterium* sp. [14], JB10Nag (accession no.: AQM74372) from *Shinella* sp. [13], Hex1 (PDB ID: 3GH4) from *Paenibacillus* sp. [19], HexA (PDB ID: 2GJX) from *H. sapiens* [20,21], SpHex (PDB ID: 1M01) from *S. plicatus*[22], and OfHex1 (PDB ID: 3NSM) from *O. furnacalis* [23,24]. Catalytic residues are labelled. P1 and P2 structures are indicated by arrows and depicted in green.

To investigate whether random coils were composed of amino acid residues at P1 and P2, structures of the GH 20 GlcNAcases were compared and shown in Figure 2. Among the GH 20 GlcNAcases, HJ5Nag had the longest coil at P1, and JB10Nag had the longest coil at P2 (Table 1; Figure 2).

## Discussion

Some studies have reported that enzyme activity can be enhanced by increasing flexibility [25–27] or reduced by decreasing flexibility, because the formation of the transition state is affected by the enzyme conformation for sampling higher-energy conformational substates[28].

A high proportion of flexible residues, such as Asp and Glu that usually have high B-factor values, are believed to cause high structural flexibility. Three mutants of *Candida antarctica* lipase B, V139E, A151D, and I255E, showed enhanced specific activity relative to that of the wild type [25,27]. Chen et al. reported that both the I368E mutation in LXYL-P1-1 and the T368E mutation in LXYL-P1-2 could increase the catalytic activity toward 7–xylosyl-10-deacetyltaxol [29,30]. Therefore, the increase in the number of amino acid residues in P1 and P2, notably Asp and Glu, may lead to an increase in catalytic activity.

Higher protein flexibility is usually accompanied by a higher percentage of random coils at the expense of the α-helix structure. Increasing the loop flexibility can also enhance the catalytic activity of the enzyme[31]. We previously calculated the ratios of random coils to α-helix structures in the seven GH 20 GlcNAcases [13,14]. The results showed that a high ratio of random coils to α-helix structures was related to a high activity in GH 20 GlcNAcases [13,14]. Therefore, the P1 structure of HJ5Nag and P2 structure of JB10Nag might be more flexible than those of most GH 20 GlcNAcases, and this increased flexibility might contribute to the higher activity of HJ5Nag and JB10Nag compared to most GH 20 GlcNAcases.

Considering that the number of amino acid residues in P2 of HJ5Nag was not greater than that of other GlcNAcases, P1 may play more important role than P2 in catalytic activity. Furthermore, structural analysis of bacterial and fungal GH 20 GlcNAcases revealed that P1 played a role in the specific binding of chitooligosaccharides[32]. Notably, E328 in P1 of OfHex1 was confirmed to be necessary for substrate binding[33].

Moreover, the charge of an amino acid residue can play a significant role in enzyme-substrate affinity, thus affecting catalytic activity. Tu et al. reported that the catalytic efficiency of endo-polygalacturonase PG8fn was improved by introducing basic Lys or Arg at position 113, as its positively charged side chain might provide a desirable environment for binding to the highly negatively charged substrate, polygalacturonic acid[34]. It is well-known that chitin and chitooligosaccharides are positively charged while other polysaccharides and oligosaccharides are negatively charged. Thus, acidic Asp and Glu with negatively charged side chains in P1 and P2 might contribute to substrate affinity, therefore improving the catalytic performance of GH 20 GlcNAcases.

In conclusion, this study proposed that these two positions, especially P1, might affect the catalytic performance of GH 20 GlcNAcases. The increase in the activity of GH 20 GlcNAcases might correspond to the enhanced structural flexibility and substrate affinity exhibited by P1 and P2. These enhancements are likely caused by increases in random coils and the number of amino acid residues, notably acidic Asp and Glu. These results are useful for guiding protein engineering to improve the catalytic activity of GH 20 GlcNAcases.
